# Association Between *OLIG2* Gene SNP rs1059004 and Negative Self-Schema Constructing Trait Factors Underlying Susceptibility to Depression

**DOI:** 10.3389/fpsyt.2021.631475

**Published:** 2021-03-08

**Authors:** Hiroshi Komatsu, Hikaru Takeuchi, Chiaki Ono, Zhiqian Yu, Yoshie Kikuchi, Yoshihisa Kakuto, Shunichi Funakoshi, Takashi Ono, Ryuta Kawashima, Yasuyuki Taki, Hiroaki Tomita

**Affiliations:** ^1^Department of Psychiatry, Tohoku University Hospital, Sendai, Japan; ^2^Miyagi Psychiatric Center, Natori, Japan; ^3^Division of Developmental Cognitive Neuroscience, Institute of Development, Aging and Cancer, Tohoku University, Sendai, Japan; ^4^Department of Disaster Psychiatry, International Research Institute of Disaster Science, Tohoku University, Sendai, Japan; ^5^Department of Community Psychiatry, Tohoku University, Sendai, Japan; ^6^Smart Ageing International Research Center, Institute of Development, Aging and Cancer, Tohoku University, Sendai, Japan; ^7^Department of Nuclear Medicine and Radiology, Institute of Development, Aging and Cancer, Tohoku University, Sendai, Japan; ^8^Division of Medical Neuroimaging Analysis, Department of Community Medical Supports, Tohoku Medical Megabank Organization, Tohoku University, Sendai, Japan; ^9^Tohoku Medical Megabank Organization, Tohoku University, Sendai, Japan; ^10^Department of Disaster Psychiatry, Graduate School of Medicine, Tohoku University, Sendai, Japan; ^11^Department of Psychiatry, Graduate School of Medicine, Tohoku University, Sendai, Japan

**Keywords:** depressive symptoms, negative self-schema, single nucleotide polymorphism, *OLIG2*, oligodendrocyte

## Abstract

Recent evidence has indicated that the disruption of oligodendrocytes may be involved in the pathogenesis of depression. Genetic factors are likely to affect trait factors, such as characteristics, rather than state factors, such as depressive symptoms. Previously, a negative self-schema had been proposed as the major characteristic of constructing trait factors underlying susceptibility to depression. Thus, the association between a negative self-schema and the functional single nucleotide polymorphism (SNP) rs1059004 in the *OLIG2* gene, which influences *OLIG2* gene expression, white matter integrity, and cerebral blood flow, was evaluated. A total of 546 healthy subjects were subjected to genotype and psychological evaluation using the Beck Depression Inventory-II (BDI-II) and the Brief Core Schema Scale (BCSS). The rs1059004 SNP was found to be associated with the self-schema subscales of the BCSS and scores on the BDI-II in an allele dose-dependent manner, and to have a predictive impact on depressive symptoms via a negative-self schema. The results suggest the involvement of a genetic factor regulating oligodendrocyte function in generating a negative-self schema as a trait factor underlying susceptibility to depression.

## Introduction

Major depressive disorder (MDD) is a common disease with a 12-month and lifetime prevalence of 6.6 and 16.2%, respectively (1). Depressive disorders, including MDD and dysthymia, are also a leading cause of the global disease burden (2). Although effective treatments (e.g., antidepressants and cognitive behavioral therapy) are available for MDD, remission rates for antidepressants are not currently optimally achieved (3). Therefore, the development of new and more effective drugs is required in the future and elucidating the pathology of MDD could help us develop these novel medicines. Depressive disorders are multifactorial diseases that are thought to develop through interactions between genetic and psychosocial factors. Although the neurobiological mechanism underlying MDD has been extensively investigated, the definitive biological mechanism remains unclear.

Prior studies based on post-mortem brain, neuroimaging, and animal studies have indicated the involvement of disrupted oligodendrocytes in the pathology of MDD. Histological examination of the post-mortem brain revealed that patients with MDD have a substantially reduced density and number of oligodendrocytes in their prefrontal cortex (4, 5). Oligodendrocyte-related genes were also found to be reduced in the temporal frontal cortex and in the white matter of the ventral prefrontal cortex in patients with MDD (6, 7). More recent studies have reported that psychological stress results in the downregulation of oligodendrocyte-related genes and reduced myelination fiber length and density in the brains of mice (8, 9). Cathomas et al. (8) revealed that after psychological stress, emotion, and microglial activity were altered in mice that were heterozygous for the cyclic nucleotide phosphodiesterase (Cnp1) oligodendrocyte gene compared to wild-type mice; this suggests that oligodendrocyte gene expression affects behavioral changes after psychosocial stress. Several diffusion tensor imaging studies have documented decreased white matter integrity in the corpus callosum and several frontal, temporal, and parietal regions in patients with MDD (10–13). In addition, other studies have found microstructural abnormalities in some fiber tracts, such as anterior callosal fibers, in patients with MDD (14, 15). Patients with MDD also show a reduced magnetic transfer ratio, which reflects demyelination in brain regions, such as the frontal and striatal regions, limbic areas, occipital white matter, and the genu and splenium of the corpus callosum (16–18). Evidence found in post-mortem brains, neuroimaging, and animal studies suggests that disrupted oligodendrocyte function may be involved in impaired mood regulation in MDD.

The *OLIG2* gene is a basic helix-loop-helix transcription factor that is expressed exclusively in oligodendrocytes and oligodendrocyte precursors and is involved in oligodendrocyte differentiation (19, 20). One post-mortem brain study found that *OLIG2* gene expression was downregulated in the temporal cortex of patients with MDD (6). The *OLIG2* gene single nucleotide polymorphism (SNP) rs1059004 is a functional SNP that influences *OLIG2* gene expression and influences white matter integrity in Caucasian populations (21–23). A more recent study revealed that SNP rs1059004 affects resting-state cerebral blood flow in a broad region of the brain as well as white matter integrity in the Japanese population (23). Genetic association studies have shown that *the OLIG2* SNP rs1059004 is associated with several psychiatric disorders such as schizophrenia and obsessive-compulsive disorder in a certain population (24–28). However, no previous studies have investigated the genetic association between this SNP and MDD.

Beck proposed the cognitive model of depression, in which negative self-schema individuals are vulnerable to developing depression in the future (29). Previous studies have shown a marked association between the schemata concerning the self and others, and the severity of depressive symptoms (30, 31). Evans et al. revealed that a negative self-schema was a risk factor for the development of depression in women (32). The results of these studies support the cognitive model of depression hypothesized by Beck (29).

Based on prior evidence indicating the involvement of oligodendrocyte abnormalities in the pathology of MDD, the present study hypothesized the association between *OLIG2* SNP rs1059007 and the negative-self core schema, as it plays a major role in constructing trait factors underlying susceptibility to MDD by influencing the function of oligodendrocytes. To verify this hypothesis, we investigated whether *the OLIG2* SNP rs1059007 was associated with self-negative core schema in healthy subjects.

## Materials and Methods

### Subjects

A total of 777 healthy, right-handed individuals were recruited, and their genotyping data, mood states, core beliefs, brain imaging data, cognitive function, aging, genetics, and daily habits were examined as detailed below and elsewhere (33–36). Of the 777 participants, all gene polymorphism data, scores on the Beck Depression Inventory-II (BDI-II), and scores on the Brief Core Schema Scale (BCSS) were successfully obtained from 546 subjects (313 men and 233 women; 20.5 ± 1.8 years of age). All subjects had normal vision and were university, college or postgraduate students, or subjects who had graduated from these institutions within 1 year prior to the experiment. None of the participants had a history of neurological or psychiatric illness. Handedness was evaluated using the Edinburgh Handedness Inventory (37). High-molecular-weight DNA was isolated from saliva specimens using Oragene containers (DNA Genotek Inc., Ottawa, Canada), according to the manufacturer's instructions. After the study procedures were fully explained, written informed consent was obtained from all participants in accordance with the Declaration of Helsinki (1991). This study was approved by the Ethics Committee of Tohoku University.

### Psychological Assessments

Participants were administered the Japanese version of the Brief Core Schema Scale (BCSS) to assess core schemas about the self and others. The BCSS is a 24-item self-report scale developed by Fowler et al. to measure core schemata with regard to the self and others (30). The BCSS includes four dimensions of self- and other-assessment: negative self, positive self, negative others, and positive others. Each dimension comprised six items that were evaluated on a five-point rating scale (0–4), and respondents were asked to indicate “yes” or “no” for whether they held each belief. If they held the belief, they were then asked to indicate the degree of their conviction on a 4-point scale ranging from 1 to 4 (1: believe slightly, 2: believe it moderately, 3: believe it very much, 4: believe it totally).

Participants were also tested using the Japanese version of the BDI-II to evaluate the degree of depressive symptoms. BDI-II, developed by Beck et al. (38), is a 21-item self-report questionnaire used to evaluate the severity of depression in normal and psychiatric subjects (39). Each item consisted of a 4-point scale from 0 (symptoms absent) to 3 (severe symptoms). The minimum and maximum BDI-II scores were 0 and 63, respectively with higher scores indicating greater severity of depressive symptoms. In normal subjects, scores above 20 have been reported to indicate depression (40).

### SNP Genotyping

Genotyping of *OLIG2* SNP rs1059004 was carried out using TaqMan assays (Applied Biosystems, Foster City, CA, USA). Polymerase chain reactions (PCRs) were performed using 20 ng of genomic DNA, 40 × TaqMan Probe Assay Mix (Probe ID: C_2442961_10) (Applied Biosystems, Waltham, MA, USA), 2 × Universal PCR Master Mix (Applied Biosystems), and nuclease-free water in a 10 μL total reaction volume; allele-specific fluorescence was measured using the CFX96 Real-Time System (Bio-Rad, Hercules, CA, USA). Information about the TaqMan Probe sequence can be obtained from https://www.thermofisher.com/order/genome-database/details/genotyping/C___2442961_20?CID=&ICID=&subtype=. The PCR cycle conditions consisted of an initial denaturation at 95°C for 10 min, followed by 50 cycles at 92°C for 15 s and at 57°C for 1 min. Then, PCR products spanning the SNP were amplified with primers (forward: gagcgctgtctggctttaac, reverse: gaggaacggccacagttcta) from two representative subjects for each of the three genotypes of this SNP and were subjected to direct sequencing to validate the TaqMan assay-based genotyping.

### Statistical Analysis

Statistical evaluations were performed using SPSS statistics 24 and Amos 23 (Japan IBM, Tokyo, Japan) software packages. Demographic variables among groups were compared using χ^2^-tests, analysis of variance, *t*-tests, or Kruskal-Wallis test, where appropriate. Deviations in genotype distribution from the Hardy-Weinberg equilibrium (HWE) were assessed using the χ^2^-test for goodness of fit. Spearman's bivariate correlation analysis was performed to examine the correlations between BDI-II scores and the four BCSS subscales. The Jonckheere-Terpstra test was performed to investigate whether *the OLIG2* SNP rs1059004 was associated with the scores on the four BCSS subscales or those of BDI-II in an allele dose-dependent manner. After the Jonckheere-Terpstra test, the *post-hoc* test was used to compare the differences between each genotype using the Bonferroni corrections.

**Figure 1:**
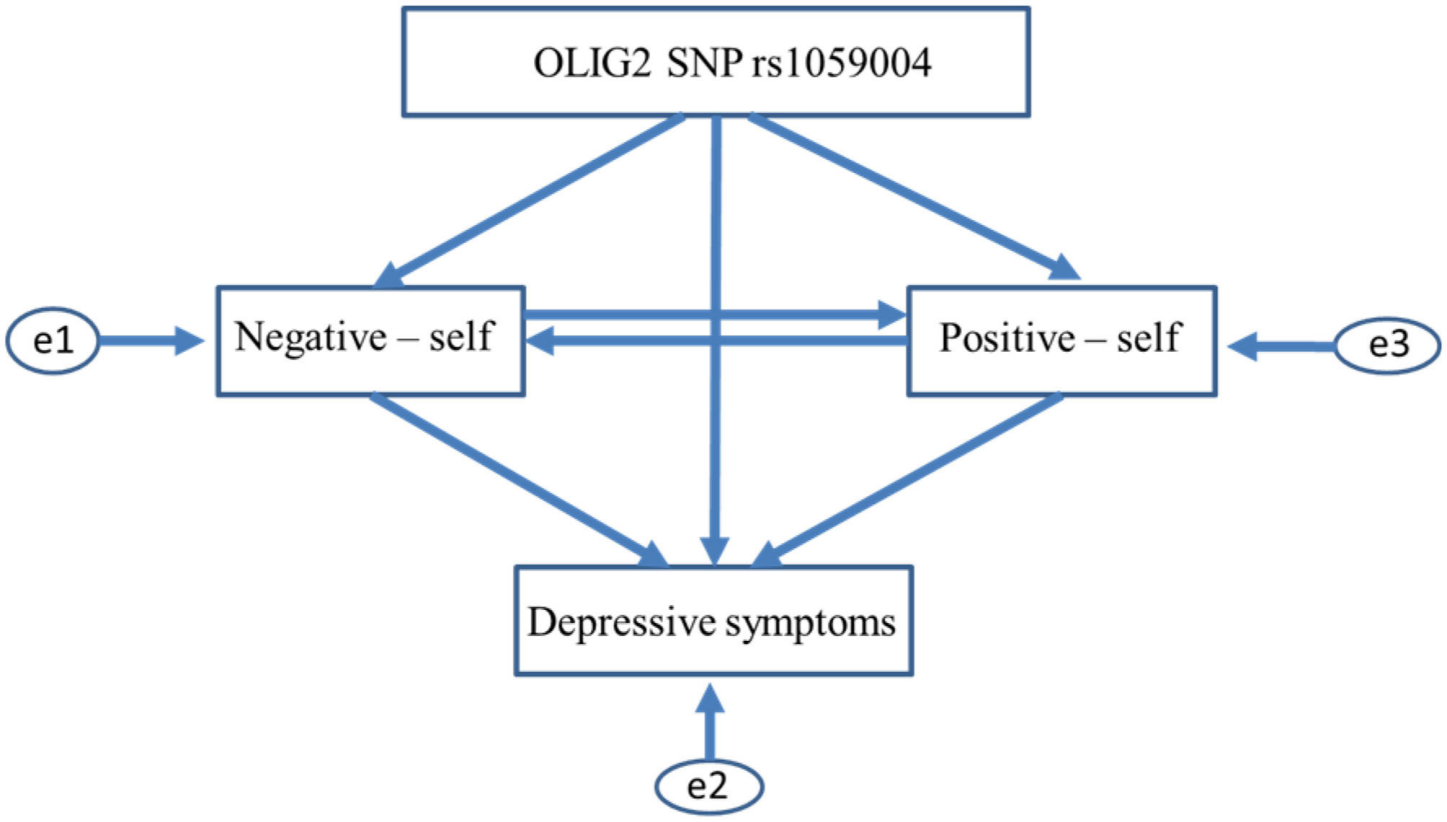
Initial path models between *OLIG2* gene SNP rs1059004, self-core schema, and depressive symptoms. The two initial path models (AIC = 20, CFI = 1, RMSEA = 0.363 in both models) shown in Figure 1 assume that *OLIG2* SNP rs1059004 affects negative self-schema (negative self), positive self-schema (positive self), and depressive symptoms in one direction. In contrast, hypothetical paths were not created among each self-schema. SNP, single nucleotide polymorphism; AIC, Akaike information criterion; CFI, comparative fit index; RMSEA, root mean square error of approximation.

The associations between *the OLIG2* SNP rs1059004, self-schema, and depressive symptoms were assessed using path analysis. Structural equation modeling was performed to evaluate the association between the above-mentioned three variables using SPSS and Amos 23, and model fits were estimated using the maximum likelihood method. The two initial hypothetical models shown in Figure 1 were constructed based on the results of the trend test, bivariate correlation analysis, and a previous study (32). In the two initial models shown in Figure 1, the self-schema affects depressive symptoms, and *OLIG2* SNP rs1059004 impacts schema and depressive symptoms in one direction. In contrast, a negative schema influences or is affected by a positive schema about the self. We removed paths scoring *p* > 0.05 from the initial models and examined whether the model fit improved. The best-fitting path model was ultimately adopted in the path analysis. Chi-square statistics were used to test the goodness-of-fit model, and the following fit indices were calculated: goodness-of-fit index (GFI), adjusted GFI (AGFI), comparative fit index (CFI), Akaike information criterion (AIC), and root mean square error of approximation (RMSEA).

To investigate whether *the OLIG2* SNP rs1059004 influenced the severity of depressive symptoms and core schema about the self and others in a gender-dependent manner, two-way analysis of covariance (ANCOVA) controlling for age, with genotype and gender as independent variables, was used to assess differences in scores on the BDI-II and on the four BCSS subscales between A-allele carriers (AA genotype + AC genotype) and non-A-allele carriers (CC genotype). As few individuals carried the AA genotype, subjects carrying the AA genotype were combined with those carrying the AC genotype, and the differences in scores on the BDI-II and BCSS subscales were compared between carriers and non-carriers of the A allele. *Post-hoc* analyses were performed using Bonferroni corrections. Statistical significance was defined as a two-tailed *p* < 0.05.

## Results

There were significant differences in age and in positive, negative, and other subscale scores between men and women (Table 1).

**Table 1:** Demographics of all variables in the healthy subjects.

	**Men (*N* = 313)**	**Women (*N =* 233)**	***t*-value**	***p*-value^**a**^**	**All subjects (*N* = 546)**
Age (mean ± sd, range)	20.7 ± 1.9, 18–27	20.3 ± 1.5, 18–26	2.622	**0.009**	20.5 ± 1.8, 18–27
BDI-II score (mean ± sd, range)	8.0 ± 6.2, 0–31	8.4 ± 6.3, 0–31	−0.78	0.436	8.2 ± 6.2, 0–31
**BCSS subscale scores**					
Negative self score (mean ± sd, range)	5.2 ± 4.4, 0–23	4.8 ± 4.3, 0–20	0.887	0.461	5.0 ± 4.3, 0–23
Positive self score (mean ± sd, range)	6.0 ± 4.3, 0–23	5.2 ± 3.8, 0–23	2.219	**0.04**	5.6 ± 4.1, 0–23
Negative other score (mean ± sd, range)	2.3 ± 3.1, 0–19	1.6 ± 2.5, 0–15	2.755	**0.003**	2.0 ± 2.9, 0–19
Positive other score (mean ± sd, range)	8.0 ± 4.4, 0–20	8.9 ± 5.0, 0–24	−2.236	**0.024**	8.4 ± 4.7, 0–24

*The table represents the mean ± sd of age and BDI-II and the BCSS scores among males and females, in addition to all other variables for all participants. The p-values and t-values from t-tests are also shown*.

*BDI-II, Beck Depression Inventory-II; BCSS, Brief Core Schema Scale; sd, standard deviation*.

a *t-test between men and women*.

The genotype distribution of the subjects was as follows: homozygous A allele (*n* = 14, 2.5%), heterozygous A/C (*n* = 155, 28.3%), and homozygous C allele (*n* = 377, 69%), which did not deviate from the HWE (χ^2^ = 0.33, *p* > 0.05). There were no significant differences in age or sex among the three *OLIG2* genotypic groups (Table 2).

**Table 2:** The demographics of subjects subdivided according to *OLIG2* genotype.

	**Genotype**			
	**C/C**	**C/A**	**A/A**	***p*-value**
N	377 (69.0%)	155 (28.3%)	14 (2.5%)	
Age (mean ± sd, range)	20.5 ± 1.8, 18–27	20.6 ± 1.8, 18–27	20.3 ± 1.6, 18–24	0.864
Gender (male/female)	221/156	87/68	5/9	0.221
BDI-II (mean ± sd, range)	11 ± 5.6, 0–31	8.9 ± 6.6, 0–31	7.9 ± 6.1, 1–20	0.03
**Scores on BCSS subscales**				
Negative self score (mean ± sd, range)	4.8 ± 4.3, 0–23	5.7 ± 4.5, 0–20	5.8 ± 4.3, 0–13	0.042
Positive self score (mean ± sd, range)	5.9 ± 4.1, 0–23	5.4 ± 4.2, 0–18	3.1 ± 3.6, 0–11	0.015
Negative other score (mean ± sd, range)	2.1 ± 3.0, 0–19	2.0 ± 2.8, 0–15	1.5 ± 2.2, 0–8	0.847
Positive other score (mean ± sd, range)	8.3 ± 4.7, 0–24	10.5 ± 4.9, 0–21	10.4 ± 4.5, 3–17	0.17

*The table shows mean ± sd for age and gender for each OLIG2 SNP rs1059004 genotype. P-values for the analysis of variance (ANOVA) are used to compare the differences in age among OLIG2 genotypes, and the chi-square tests between gender and OLIG2 genotype were represented in the table. The table shows p-values for the Kruskal-Wallis test to compare the difference in BDI-II and BCSS subscales among OLIG2 genotypes*.

*sd, standard deviation*.

Meanwhile, the Kruskal-Wallis test showed significant differences in BDI-II and the negative and positive self subscales of the BCSS among *OLIG2* genotypes (*p* = 0.03, *p* = 0.042, *p* = 0.015, respectively, Table 2). The Cronbach's α values for the BDI-II total score, the negative and positive self subscale scores, and the negative and positive other subscale scores were 0.82, 0.80, 0.82, 0.78, and 0.86, respectively.

### Bivariate Associations Between BDI-II and Four BCSS Subscale Scores

Spearman's bivariate correlation analysis revealed a positive association between BDI-II score and the negative self and negative other subscales of the BCSS (*r* = 0.636 and *r* = 0.296, respectively, *p* < 0.001, Table 3). There was a significant negative association between BDI-II score and the positive self and positive other subscales of the BCSS (*r* = −0.459 and *r* = −0.306, respectively, *p* < 0.001, Table 3).

**Table 3:** Spearman's bivariate correlation analysis of age, BDI-II score, and BCSS scores.

	**Age**	**BDI-II**	**Negative self**	**Positive self**	**Negative other**	**Positive other**
Age	–					
BDI-II	−0.057	–				
Negative self	−0.062	0.636^**^	–			
Positive self	0.096	−0.459^**^	−0.404^**^	–		
Negative other	−0.033	0.296^**^	0.357^**^	0.009	–	
Positive other	−0.050	−0.306^**^	−0.202^**^	0.296^**^	−0.349^**^	–

*The table exhibits Spearman's correlation coefficients between age and scores on the BDI-II and the four BCSS subscales*.

*BDI-II, Beck Depression Inventory-II; BCSS, Brief Core Schema Scale*.

** *Bonferroni-corrected p < 0.001*.

### Association Between OLIG2 SNP rs1059004 and the Severity of Depressive Symptoms

**Figure 2:**
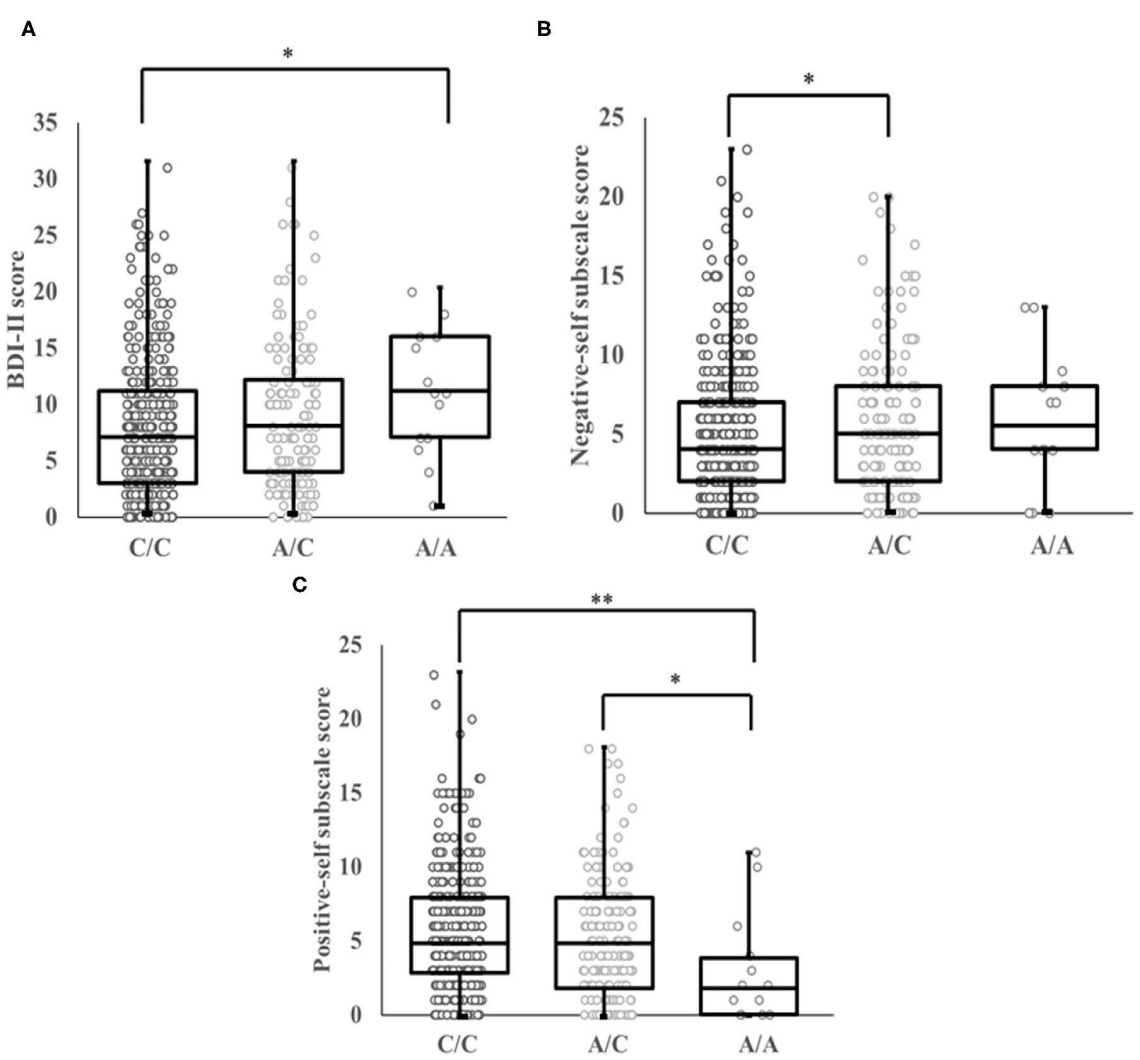
**(A)** Association between genotype at SNP rs1059004 in the *OLIG2* gene and BDI-II scores. There were significant correlations between A allele dosage and BDI-II scores among the three genotype groups. BDI-II scores decreased in a C-allele dose-dependent manner (*p* = 0.022, Jonckheere-Terpstra test). AA genotype carriers had a significantly higher BDI-II score than the CC genotype carriers (Bonferroni-corrected *p* = 0.041). **(B)** Association between genotype at SNP rs1059004 in the *OLIG2* gene and negative-self subscale scores. Significant gene-dose associations between the SNP rs1089004 and negative-self subscale scores were observed among the three genotype groups. Negative-self subscale scores decreased in a C-allele dose-dependent manner (*p* = 0.012, Jonckheere-Terpstra test). Subjects with AC genotype had a significantly higher negative-self subscale score compared to those with CC genotype (*p* = 0.026). **(C)** Association between genotype at SNP rs1059004 and positive-self subscale scores. The Jonckheere-Terpstra test also showed significant associations between A allele dosage and positive-self subscale scores among the three genotype groups. Positive-self subscale scores increased in a C-allele dose-dependent manner (*p* = 0.037, Jonckheere-Terpstra test). People carrying the AA genotype had a significantly lower positive-self subscale score than those with the CC genotype and AC genotype, respectively (Bonferroni-corrected *p* = 0.009, *p* = 0.049). The bars of the box plot show the range from minimum scores to maximum scores. SNP, single nucleotide polymorphism. *p < 0.05, **p < 0.01 compared to the CC genotype.

The present study found a significant association between *the OLIG2* SNP rs1059004 and BDI-II score in an allele dose-dependent manner (Jonckheere-Terpstra test, *p* = 0.022, Figure 2A). *Post-hoc* analysis showed that AA genotype carriers had a significantly higher BDI-II score than the CC genotype carriers (Bonferroni-corrected *p* = 0.041, Figure 2A). Two-way ANCOVA adjusted for age, with genotype and gender as the fixed factors showed that there was a significant main effect of genotype on BDI-II score (Table 4). However, the genotype-gender interaction was not significant for the BDI-II score (Table 4).

**Table 4:** The results of two-way ANCOVA controlling for age, with genotype and gender as independent variables.

	**Men**		**Women**		**Genotype**		**Gender**		**Genotype × ** **Gender**	
	**Non-A-allele carriers (CC genotype)**	**A-allele carriers (AA genotype +AC genotype)**	**Non-A-allele carriers (CC genotype)**	**A-allele carriers (AA genotype +AC genotype)**	***F***	***p***	***F***	***p***	***F***	***p***
N	221	92	156	77						
BDI-II (mean ± sd)	7.88 ± 6.03	8.48 ± 6.63	7.86 ± 6.30	9.74 ± 6.28	4.569	**0.033**	0.889	0.346	1.219	0.27
**Scores on subscales of BCSS**										
Negative self schema score (mean ± sd)	4.98 ± 4.33	5.79 ± 4.61	4.51 ± 4.29	5.62 ± 4.41	5.626	**0.018**	0.81	0.368	0.131	0.718
Positive self schema score (mean ± sd)	6.18 ± 4.28	5.59 ± 4.52	5.40 ± 3.78	4.82 ± 3.81	2.449	0.118	2.988	0.084	0	0.998
Negative other schema score (mean ± sd)	2.41 ± 3.20	2.09 ± 2.99	1.56 ± 2.56	1.75 ± 2.42	0.055	0.815	5.444	**0.02**	0.911	0.34
Positive other schema score (mean ± sd)	7.79 ± 4.39	8.73 ± 4.69	8.92 ± 5.05	9.13 ± 5.18	1.722	0.19	2.492	0.115	0.664	0.416

*The table displays differences in the scores on the BDI-II and the BCSS by gender between A-allele carriers and non-A allele carriers. F-values and p-values for the main effect of genotype, gender and their interaction effect are also represented in the table*.

*BDI-II, Beck Depression Inventory-II; BCSS, Brief Core Schema Scale; sd, standard deviation*.

### Association Between OLIG2 SNP rs1059004 and Core Schema About the Self and Others

The Jonckheere-Terpstra test showed that *the OLIG2* SNP rs1059004 was associated with both negative and positive self-schema in an allele dose-dependent manner (Jonckheere-Terpstra test, *p* = 0.012, *p* = 0.037; Figures 2B,C). Subjects with the AC genotype had a significantly higher negative-self subscale score than those with the CC genotype (Bonferroni-corrected *p* = 0.026, Figure 2B). Individuals carrying the AA genotype had a significantly lower positive-self subscale score than those with the CC genotype and AC genotype, respectively (Bonferroni-corrected *p* = 0.009, *p* = 0.049, Figure 2C).

Two-way ANCOVA controlling for age and with genotype and gender as the fixed factors showed that there was a significant main effect of genotype on negative-self subscale score (Table 4). However, no significant genotype-gender interactions were observed for scores on the four BCSS subscales (Table 4).

### Path Analysis of the Relationship Between OLIG2 rs1059004, the Self-Core Schema, and Depressive Symptoms

**Figure 3:**
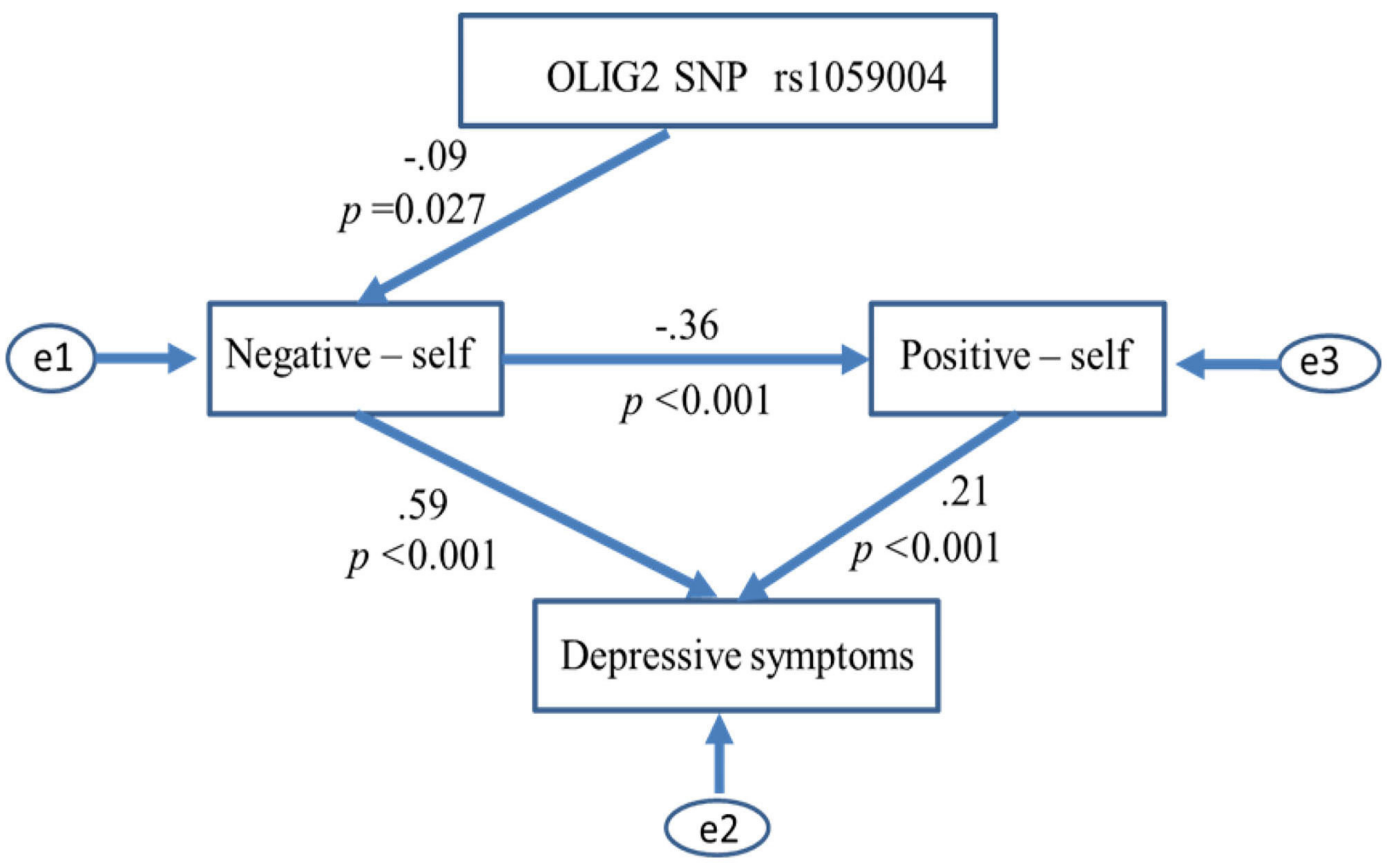
Relationships between *OLIG2* SNP rs1059004, self-core schema and depressive symptoms. Paths scoring *p* > 0.05 were removed from the initial models, and the best-fitting path model was ultimately adopted in the path analysis. The path model produced the best model fit results, as shown below: Chi-square = 2.669, df = 2, P = 0.263; GFI = 0.998; AGFI = 0.988; CFI = 0.998; AIC = 18.669; and RMSEA = 0.025. This path model indicates the predictive effect of *the OLIG2* SNP rs1059004 on depressive symptoms via a negative self-schema (negative self) that influences a positive self-schema (positive self). SNP, single nucleotide polymorphism; GFI, goodness-of-fit index; AGFI, adjusted GFI; CFI, comparative fit index; AIC, Akaike information criterion; RMSEA, root mean square error of approximation.

We removed paths scoring *p* > 0.05 from the initial models (AIC = 20, CFI = 1, RMSEA = 0.363 in both models) and examined whether the model fit improved. Ultimately, the path model shown in Figure 3 produced the best results in the model fit evaluations (Chi-square = 2.669, df = 2, *P* = 0.263; GFI = 0.998; AGFI = 0.988; CFI = 0.998; AIC = 18.669; RMSEA = 0.025). The number of C alleles of the *OLIG2* gene SNP rs1059004 was negatively associated with the negative self-schema, and the schema was assumed to affect the positive self-schema and lead to a higher severity of depressive symptoms (Figure 3).

## Discussion

Based on prior evidence that the disruption of oligodendrocytes may be implicated in the pathology of MDD, the present study hypothesized an association between the *OLIG2* SNP rs1059004 and the negative self-schema, constructing trait factors underlying susceptibility to MDD. To verify this hypothesis, the associations between *the OLIG2* SNP rs1059004, BCSS and BDI-II scores were examined in 546 healthy subjects. Consistent with the above hypothesis, the number of C alleles in *the OLIG2* gene SNP rs1059004 was associated with decreased BDI-II scores and the negative BCSS self-schema subscale in an allele dose-dependent manner. Path analysis revealed that a negative self-schema mediated the association between *the OLIG2* SNP rs1059004 and the severity of depressive symptoms. The results of the present study indicate that *the OLIG2* SNP rs1059004 has a predictive impact on depressive symptoms via a negative schema of the self. In addition, the results suggest the involvement of a genetic factor regulating oligodendrocyte function in generating a negative-self schema that plays a major role in constructing factors underlying susceptibility to depression.

Although previous studies have not found significant differences in scores for any BCSS subscale between men and women (30, 31), the current study found that gender created significant differences in positive, negative, and “other” BCSS subscale scores. This discrepancy in the results may be due to differences in the ethnicities, number, and/or ages of the participants. The findings of the present study indicate that the positive self-schema may be affected by a variety of experiences during young adulthood in the Japanese population. In contrast, four of the BCSS subscales were significantly correlated with the severity of depressive symptoms, consistent with previous reports (30, 31).

Previous genetic association studies have exclusively focused on genes related to monoamines, the hypothalamic-pituitary-adrenal axis, and glutamatergic neurotransmitters. For example, polymorphisms of the glucocorticoid receptor gene, monoamine oxidase A gene, and group-2 metabotropic glutamate receptor gene have been previously reported to be associated with MDD (41–44). Although polymorphisms of oligodendrocyte-related genes have been shown to be associated with schizophrenia (24), no previous studies have investigated the genetic association between the polymorphisms of oligodendrocyte-related genes and MDD. This is the first study to reveal that *the OLIG2* SNP rs1059004 was associated with the severity of depressive symptoms and with negative self-schema. Whether oligodendrocyte disruption is a causative factor in mood dysregulation remains unclear, but several studies have suggested that changes in oligodendrocyte function and structure can influence neural circuits that mediate mood regulation in mice (8, 45–47). For example, mice exposed to cuprizone, a mouse model of demyelination, showed deceased anxiety-like behavior. Moreover, mice lacking the Cnp1 oligodendrocyte-related gene were found to show depressive-like behaviors (45). Therefore, *the OLIG2* SNP rs1059004 may be associated with the severity of depressive symptoms and negative self-schema by influencing the function of oligodendrocytes. One functional magnetic resonance imaging-based study indicated that the activities of the anterior cingulate cortex and the inferior frontal cortex are involved in negative self-reflection (48), and prior investigations have found that the *OLIG2* polymorphism rs1059004 affects not only white matter integrity but also resting-state cerebral blood flow in widespread brain regions, including the anterior cingulate and inferior frontal regions (23). Therefore, the effect of SNP rs1059004 on brain perfusion may be a biological mechanism underlying the association between the variant and negative self-schema. Further studies are needed to reveal the biological basis of the association between *the OLIG2* SNP rs1059004 and negative self-schema.

Beck proposed the cognitive model of depression, in which individuals who hold negative self-schemas are vulnerable to developing depression in the future (29). Evans et al. revealed that a negative self-schema was a risk factor for the onset of depression in women in a longitudinal study (32). This finding may support the cognitive model of depression proposed by Beck. Therefore, negative self-schema could play a major role in constructing trait factors underlying susceptibility to MDD. The results of the present study indicate a significant association between *the OLIG2* SNP rs1059004 and a negative self-schema, constructing trait factors concerning susceptibility to MDD. Considering the significant association between the *OLIG2* SNP rs1059004 and a negative self-schema as the trait factor underlying the development of depression, it would be worthwhile to conduct a longitudinal study investigating whether SNP rs1059004 affects the risk of the onset of MDD by influencing negative self-schema.

The present study has several limitations. First, the participants were limited in terms of age and education history; that is, only young adults and university, college, or postgraduate students were examined; therefore, the results of the present study may not apply to the general population. The second limitation is the cross-sectional nature, which cannot disentangle the direction of effects between variables. The third limitation is that some problems have recently arisen regarding single-gene studies of multifactorial phenotypes, such as depression, and many studies have failed to be replicable. For example, Culverhouse et al. found no proof that the thousands of studies on the relationship between 5-HTTLPR and depression provided evidence of a genuine genetic difference (49). More recently, Border et al. found no evidence for “any candidate gene polymorphism associations with depression phenotypes or any polymorphism-by-environment moderator effects (50).” Although a negative self-schema can relatively reflect trait factors compared with depression, the phenotype is also highly polygenic, and the genetic effect expected from a single gene can be small. However, in the present study, the number of participants was small, the AA genotype was relatively low compared to other genotypes, the power was insufficient, and cross-validation was not performed. Therefore, it will be necessary to replicate these results with a much larger sample size in the future. The fourth limitation is that it is not clear whether the genetic association in the present study is applied to ethnicities other than Japanese. The fifth limitation is that although prior studies indicated that SNP rs1059004 predicted OLIG2 gene expression in Caucasian subjects, it is unclear whether a significant association exists between the SNP rs1059004 and OLIG2 gene expression in Japanese subjects. This study is the first to indicate a significant genetic association between the *OLIG2* SNP rs1059004 and negative self-schema as a trait factor in susceptibility to MDD in Japanese subjects. The results of the present study suggest an association between *the OLIG2* SNP rs1059004 and negative self-schema by influencing the function of oligodendrocytes. These results may support the involvement of a genetic factor regulating oligodendrocyte function in generating a negative self-schema as a trait factor underlying susceptibility to depression in the pathology of MDD.

## Data Availability Statement

The SNP data presented in the study are publicly available. This data can be found in dbSNP (https://www.ncbi.nlm.nih.gov/SNP/snp_viewTable.cgi?handle=TOHOKUPSY).

## Ethics Statement

The studies involving human participants were reviewed and approved by The Ethics Committee of Tohoku University. The patients/participants provided their written informed consent to participate in this study.

## Author Contributions

HK, HTa, YKi, CO, ZY, and HTo contributed to the acquisition of data or the analysis and interpretation of data. HK and HTa were involved in drafting the manuscript. HTa, YKa, SF, TO, and HTo critically revised the manuscript for important scientific content. HTa, RK, YT, and HTo made substantial contributions to the conception and design of the study. All authors read and approved the final version of the manuscript and agreed on the order in which their names would be listed in the manuscript.

## Conflict of Interest

The authors declare that the research was conducted in the absence of any commercial or financial relationships that could be construed as a potential conflict of interest.

## References

[1] Kessler RC Berglund P Demler O Jin R Koretz D Merikangas KR. The epidemiology of major depressive disorder: results from the National Comorbidity Survey Replication (NCS-R). JAMA. (2003) 289:3095–105. 10.1001/jama.289.23.3095 12813115

[2] Ferrari AJ Charlson FJ Norman RE Patten SB Freedman G Murray CJ. Burden of depressive disorders by country, sex, age, and year: findings from the global burden of disease study 2010. PLOS Med. (2013) 10:e1001547. 10.1371/journal.pmed.1001547 24223526 PMC3818162

[3] Rush AJ Trivedi MH Wisniewski SR Nierenberg AA Stewart JW Warden D. Acute and longer-term outcomes in depressed outpatients requiring one or several treatment steps: a STAR^*^D report. Am J Psychiatry. (2006) 163:1905–17. 10.1176/ajp.2006.163.11.1905 17074942

[4] Hayashi Y Tatebayashi Y. A flow cytometric postmortem brain study for major depressive disorders: implication for oligodendroglial differentiation and functions. Nihon Shinkei Seishin Yakurigaku Zasshi. (2012) 32:211–8. 23012889

[5] Vostrikov VM Uranova NA Orlovskaya DD. Deficit of perineuronal oligodendrocytes in the prefrontal cortex in schizophrenia and mood disorders. Schizophr Res. (2007) 94:273–80. 10.1016/j.schres.2007.04.014 17566708

[6] Aston C Jiang L Sokolov BP. Transcriptional profiling reveals evidence for signaling and oligodendroglial abnormalities in the temporal cortex from patients with major depressive disorder. Mol Psychiatry. (2005) 10:309–22. 10.1038/sj.mp.4001565 15303102

[7] Rajkowska G Mahajan G Maciag D Sathyanesan M Iyo AH Moulana M. Oligodendrocyte morphometry and expression of myelin - related mRNA in ventral prefrontal white matter in major depressive disorder. J Psychiatr Res. (2015) 65:53–62. 10.1016/j.jpsychires.2015.04.010 25930075 PMC4836860

[8] Cathomas F Azzinnari D Bergamini G Sigrist H Buerge M Hoop V. Oligodendrocyte gene expression is reduced by and influences effects of chronic social stress in mice. Genes Brain Behav. (2019) 18:e12475. 10.1111/gbb.12475 29566304

[9] Lehmann ML Weigel TK Elkahloun AG Herkenham M. Chronic social defeat reduces myelination in the mouse medial prefrontal cortex. Sci Rep. (2017) 7:46548. 10.1038/srep46548 28418035 PMC5394533

[10] de Diego-Adeliño J Pires P Gómez-Ansón B Serra-Blasco M Vives-Gilabert Y Puigdemont D. Microstructural white-matter abnormalities associated with treatment resistance, severity and duration of illness in major depression. Psychol Med. (2014) 44:1171–82. 10.1017/S003329171300158X 23962469

[11] Jiang J Zhao YJ Hu XY Du MY Chen ZQ Wu M. Microstructural brain abnormalities in medication-free patients with major depressive disorder: a systematic review and meta-analysis of diffusion tensor imaging. J Psychiatry Neurosci. (2017) 42:150–63. 10.1503/jpn.150341 27780031 PMC5403660

[12] Ma N Li L Shu N Liu J Gong G He Z. White matter abnormalities in first-episode, treatment-naive young adults with major depressive disorder. Am J Psychiatry. (2007) 164:823–6. 10.1176/ajp.2007.164.5.823 17475743

[13] Matsuoka K Yasuno F Kishimoto T Yamamoto A Kiuchi K Kosaka J. Microstructural differences in the corpus callosum in patients with bipolar disorder and major depressive disorder. J Clin Psychiatry. (2017) 78:99–104. 10.4088/JCP.15m09851 27574839

[14] Osoba A Hänggi J Li M Horn DI Metzger C Eckert U. Disease severity is correlated to tract specific changes of fractional anisotropy in MD and CM thalamus–a DTI study in major depressive disorder. J Affect Disord. (2013) 149:116–28. 10.1016/j.jad.2012.12.026 23489404

[15] Yamada S Takahashi S Ukai S Tsuji T Iwatani J Tsuda K. Microstructural abnormalities in anterior callosal fibers and their relationship with cognitive function in major depressive disorder and bipolar disorder: a tract-specific analysis study. J Affect Disord. (2015) 174:542–8. 10.1016/j.jad.2014.12.022 25556672

[16] Chen Z Zhang H Jia Z Zhong J Huang X Du M. Magnetization transfer imaging of suicidal patients with major depressive disorder. Sci Rep. (2015) 5:9670. 10.1038/srep09670 25853872 PMC4389668

[17] Gunning-Dixon FM Hoptman MJ Lim KO Murphy CF Klimstra S Latoussakis V. Macromolecular white matter abnormalities in geriatric depression: a magnetization transfer imaging study. Am J Geriatr Psychiatry. (2008) 16:255–62. 10.1097/JGP.0000300628.33669.03 18378551

[18] Kumar A Gupta RC Albert Thomas M Alger J Wyckoff N Hwang S. Biophysical changes in normal-appearing white matter and subcortical nuclei in late-life major depression detected using magnetization transfer. Psychiatry Res. (2004) 130:131–40. 10.1016/j.pscychresns.2003.12.002 15033183

[19] Ligon KL Fancy SP Franklin RJ Rowitch DH. Olig gene function in CNS development and disease. Glia. (2006) 54:1–10. 10.1002/glia.20273 16652341

[20] Meijer DH Kane MF Mehta S Liu H Harrington E Taylor CM. Separated at birth? The functional and molecular divergence of OLIG1 and OLIG2. Nat Rev Neurosci. (2012) 13:819–31. 10.1038/nrn3386 23165259 PMC3733228

[21] Mitkus SN Hyde TM Vakkalanka R Kolachana B Weinberger DR Kleinman JE. Expression of oligodendrocyte-associated genes in dorsolateral prefrontal cortex of patients with schizophrenia. Schizophr Res. (2008) 98:129–38. 10.1016/j.schres.2007.09.032 17964117 PMC2259271

[22] Prata DP Kanaan RA Barker GJ Shergill S Woolley J Georgieva L. Risk variant of oligodendrocyte lineage transcription factor 2 is associated with reduced white matter integrity. Hum Brain Mapp. (2013) 34:2025–31. 10.1002/hbm.22045 22505278 PMC6870420

[23] Komatsu H Takeuchi H Kikuchi Y Ono C Yu Z Iizuka K. Ethnicity-dependent effects of schizophrenia risk variants of the OLIG2 gene on OLIG2 transcription and white matter integrity. Schizophr Bull. (2020) 46:1619–28. 10.1093/schbul/sbaa049 32285113 PMC7846078

[24] Georgieva L Moskvina V Peirce T Norton N Bray NJ Jones L. Convergent evidence that oligodendrocyte lineage transcription factor 2 (OLIG2) and interacting genes influence susceptibility to schizophrenia. Proc Natl Acad Sci USA. (2006) 103:12469–74. 10.1073/pnas.0603029103 16891421 PMC1567903

[25] Huang K Tang W Tang R Xu Z He Z Li Z. Positive association between OLIG2 and schizophrenia in the Chinese Han population. Hum Genet. (2008) 122:659–60. 10.1007/s00439-007-0434-z 17934761

[26] Sims R Hollingworth P Moskvina V Dowzell K O'Donovan MC Powell J. Evidence that variation in the oligodendrocyte lineage transcription factor 2 (OLIG2) gene is associated with psychosis in Alzheimer's disease. Neurosci Lett. (2009) 461:54–9. 10.1016/j.neulet.2009.05.051 19477230

[27] Stewart SE Platko J Fagerness J Birns J Jenike E Smoller JW. A genetic family-based association study of OLIG2 in obsessive-compulsive disorder. Arch Gen Psychiatry. (2007) 64:209–14. 10.1001/archpsyc.64.2.209 17283288

[28] Zhang X Liu J Guo Y Jiang W Yu J. populationAssociation. Depress Anxiety. (2015) 32:720–7. 10.1002/da.22394 26271930

[29] Beck AT. Thinking and depression. ii. theory and therapy. Arch Gen Psychiatry. (1964) 10:561–71. 10.1001/archpsyc.1964.01720240015003 14159256

[30] Fowler D Freeman D Smith B Kuipers E Bebbington P Bashforth H. The Brief Core Schema Scales (BCSS): psychometric properties and associations with paranoia and grandiosity in non-clinical and psychosis samples. Psychol Med. (2006) 36:749–59. 10.1017/S0033291706007355 16563204

[31] Uchida T Kawamura C Mifune N Hamaie Y Matsumoto K Ambo H. The Japnanese version of the brief core schema scale for schemata concerning the self and others: indentification of Shema patterns and relationschip with depression. Jpn J Pers. (2012) 20:143–54. 10.2132/personality.20.143

[32] Evans J Heron J Lewis G Araya R Wolke D ALSPAC Study Team. Negative self-schemas and the onset of depression in women: longitudinal study. Br J Psychiatry. (2005) 186:302–7. 10.1192/bjp.186.4.302 15802686

[33] Takeuchi H Taki Y Nouchi R Hashizume H Sekiguchi A Kotozaki Y. Anatomical correlates of self-handicapping tendency. Cortex. (2013) 49:1148–54. 10.1016/j.cortex.2013.01.014 23465364

[34] Takeuchi H Taki Y Nouchi R Hashizume H Sekiguchi A Kotozaki Y. Effects of working memory training on functional connectivity and cerebral blood flow during rest. Cortex. (2013) 49:2106–25. 10.1016/j.cortex.2012.09.007 23079491

[35] Takeuchi H Taki Y Nouchi R Sekiguchi A Kotozaki Y Miyauchi CM. Regional gray matter density is associated with achievement motivation: evidence from voxel-based morphometry. Brain Struct Funct. (2014) 219:71–83. 10.1007/s00429-012-0485-3 23212300 PMC3889816

[36] Takeuchi H Taki Y Sassa Y Hashizume H Sekiguchi A Fukushima A. Brain structures associated with executive functions during everyday events in a non-clinical sample. Brain Struct Funct. (2013) 218:1017–32. 10.1007/s00429-012-0444-z 22851058 PMC3695328

[37] Oldfield RC. The assessment and analysis of handedness: the Edinburgh inventory. Neuropsychologia. (1971) 9:97–113. 10.1016/0028-3932(71)90067-4 5146491

[38] Beck AT Ward CH Mendelson M Mock J Erbaugh J. An inventory for measuring depression. Arch Gen Psychiatry. (1961) 4:561–71. 10.1001/archpsyc.1961.01710120031004 13688369

[39] Beck AT Steer RA Brown GK. Manual for the Beck Depression Inventory - II. San Antonio, TX: The Psychological Corporation (1996) 10.1037/t00742-000

[40] Kendall PC Hollon SD Beck AT Hammen CL Ingram RE. Issues and recommendations regarding use of the beck depression inventory. Cognit Ther Res. (1987) 11:289–99. 10.1007/BF01186280

[41] Fan M Liu B Jiang T Jiang X Zhao H Zhang J. Meta-analysis of the association between the monoamine oxidase-A gene and mood disorders. Psychiatr Genet. (2010) 20:1–7. 10.1097/YPG.0b013e3283351112 20010318

[42] Tsunoka T Kishi T Ikeda M Kitajima T Yamanouchi Y Kinoshita Y. Association analysis of group II metabotropic glutamate receptor genes (GRM2 and GRM3) with mood disorders and fluvoxamine response in a Japanese population. Prog Neuropsychopharmacol Biol Psychiatry. (2009) 33:875–9. 10.1016/j.pnpbp.2009.04.007 19386277

[43] van Rossum EF Binder EB Majer M Koper JW Ising M Modell S. Polymorphisms of the glucocorticoid receptor gene and major depression. Biol Psychiatry. (2006) 59:681–8. 10.1016/j.biopsych.2006.02.007 16580345

[44] Yoon HK Kim YK. Association between glycogen synthase kinase-3beta gene polymorphisms and major depression and suicidal behavior in a Korean population. Prog Neuropsychopharmacol Biol Psychiatry. (2010) 34:331–4. 10.1016/j.pnpbp.2009.12.009 20015462

[45] Edgar NM Touma C Palme R Sibille E. Resilient emotionality and molecular compensation in mice lacking the oligodendrocyte-specific gene Cnp1. Transl Psychiatry. (2011) 1:e42. 10.1038/tp.2011.40 22832658 PMC3309485

[46] Franco-Pons N Torrente M Colomina MT Vilella E. Behavioral deficits in the cuprizone-induced murine model of demyelination/remyelination. Toxicol Lett. (2007) 169:205–13. 10.1016/j.toxlet.2007.01.010 17317045

[47] Xu H Yang HJ Zhang Y Clough R Browning R Li XM. Behavioral and neurobiological changes in C57BL/6 mice exposed to cuprizone. Behav Neurosci. (2009) 123:418–29. 10.1037/a0014477 19331464

[48] Ma Y Li B Wang C Shi Z Sun Y Sheng F. 5-HTTLPR polymorphism modulates neural mechanisms of negative self-reflection. Cereb Cortex. (2014) 24:2421–9. 10.1093/cercor/bht099 23588187

[49] Culverhouse RC Saccone NL Horton AC Ma Y Anstey KJ Banaschewski T. Collaborative meta-analysis finds no evidence of a strong interaction between stress and 5-HTTLPR genotype contributing to the development of depression. Mol Psychiatry. (2018) 23:133–42. 10.1038/mp.2017.44 28373689 PMC5628077

[50] Border R Johnson EC Evans LM Smolen A Berley N Sullivan PF. No support for historical candidate gene or candidate gene-by-interaction hypotheses for major depression across multiple large samples. Am J Psychiatry. (2019) 176:376–87. 10.1176/appi.ajp.2018.18070881 30845820 PMC6548317

